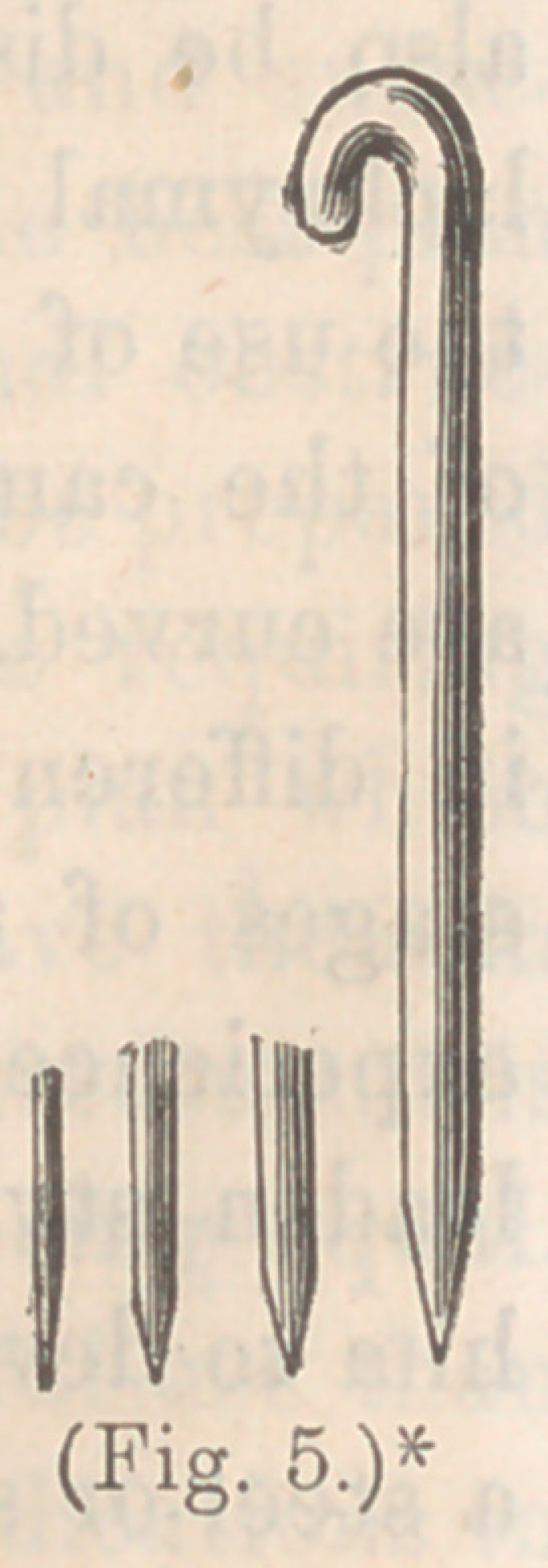# Old and New Methods of Treating Lachrymal Obstructions

**Published:** 1870-10

**Authors:** H. W. Boyd

**Affiliations:** Prof. of Anatomy, Chicago Medical College


CHICAGO MEDICAL EXAMINER.

N. S. DAVIS, M.D., Editor.

VOL. XI. OCTOBER, 1870. NO. 10.

### Original Contributions.

ARTICLE XXX.

### OLD AND NEW METHODS OF TREATING LACHRYMAL OBSTRUCTIONS.

By H. W. BOYD, M.D., Prof. of Anatomy, Chicago Medical College.

The lachrymal apparatus consists of the lachrymal gland, which is the source of the lachrymal fluid, and the canaliculi, lachrymal sac, and nasal duct, or the passages which conduct the tears from the eye to the nose. The lachrymal gland is situated in the lachrymal fossa, a depression in the orbital plate of the frontal bone; its ducts are from six to twelve in number, each about the size of a hair. They open into the sac of the conjunctiva, at the outer upper-third of the orbit. The canaliculi are canals, about three or four lines in length, and less than one-third of a line in calibre. It consists of a fine, pale, hard, mucous membrane, containing a few mucous glands and pavement epithelium. They begin near the inner extremity of each lid, at the small prominence near the caruncle. The commencement of a canaliculus is called the punctum. From this point they pass along, near the margin of the lid, the lower one passing upwards and inwards, and the upper one downwards and inwards, until they unite and open by one orifice into the lachrymal sac. Their course, in health, is free, opening into the sac without valves.

The tears consist of "pure water, mixed with a small amount of salt and albumen."

The constant motion of the lids in winking causes the tears to be equally distributed over the convexity of the globe, and thus serves to lubricate the anterior surface of the organ, as well as to wash away foreign substances, such as particles of dust, from the cornea; and, owing to the peculiar arrangement of the parts, the tears naturally flow towards the inner canthus, and are forced by the lids through the contraction of the orbicularis muscle, through the puncta into the canaliculi, and on into the lachrymal sac, thence through the nasal duct into the nose.

(Fig. 1.)*

Now, it is evident, that any obstruction of the canaliculi, or nasal duct, must interfere with the passage of the tears, and, as they are continually being secreted by the lachrymal gland, they will collect in the sac of the conjunctiva, and, finding no outlet, will fall over the lid and run down the cheek, or, by collecting in the eye, will very greatly interfere with vision. Besides this watery condition of the eye, there will be dryness of the nostril, from the absence of the tears. These obstructions may exist either in the canaliculi or nasal duct; they are caused by deposits of lymph, cicatrices, inflammation, redundancy of mucous membrane, etc. Many methods have been devised, at different times, for the cure of these obstructions, and, even to-day, the plans of treatment differ very materially with different surgeons. In inflammation of the lachrymal sac, it was the practice of the older surgeons to lay open the entire sac by an incision through the external integuments, or to destroy it with the hot iron or escharotics; but this practice is now almost obsolete, having been superseded by improved methods of treatment; and it is a question with the most learned ophthalmologists whether it is ever necessary or advisable, in the

* Lachrymal Apparatus.—A. Punctua lachrymalia. B. Canaliculi. C. Lachrymal sac. D. Nasal duct. E. Lachrymal gland, showing its ducts.

present light of science, to lay open the lachrymal sac by external incision, or to destroy it by escharotics, as was practiced a few years since, either in inflammation of the sac or stricture of the nasal duct. It is believed that we are now acquainted with methods of treatment, to which the most obstinate cases of stricture of the nasal duct, or chronic inflammation of the sac must, in the course of time, succumb. In abscess of the lachrymal sac, the pus should be evacuated through the canaliculi, either by slight pressure upon the sac with the thumb or finger, or, if it cannot be accomplished in this way, a small-grooved probe should be introduced through the punctum and canaliculus into the sac, and, by slight pressure, the pus will escape through the groove in the probe; or, if this does not give it vent with sufficient freedom, then the punctum may be slit up, for a short distance, either with a pair of fine scissors, or it may be slit up in the small-grooved probe with a small, sharp knife;—as a rule, it will be sufficient simply to introduce the probe or small-grooved director. Strictures of the nasal duct were formerly treated by means of styles made of silver or lead, and introduced into the duct by means of an external opening through the integument.

These styles were introduced by means of a slender, double-edged knife, with a groove on one side of the blade. This knife was introduced through the external integument, near the inner canthus of the lids, into the opening of the nasal duct. The style is then introduced and forced through the opening, guided by the groove in the blade of the knife, until the lower extremity of the style rests upon the floor of the nostril; the knife is then withdrawn, and the style, which has a head or hook at its upper extremity, is permitted to remain, and the tears would escape through the duct, by the side of the style. A method, devised by Dupeytren, was to introduce a silver tube, and thus procure an outlet for the tears. This tube was designed to be permanent; an external incision was made, the tube arranged in position to conduct the tears into the nose, and the opening allowed to heal up. Cases are reported, in which these tubes remained in position for years, but, as a rule, they soon become displaced. They would either slip up or down, or get out of position in some way, or would cause such irritation and pain as to necessitate their removal. A modification of this plan was made use of by Mr. Walthen, an English surgeon, in which the design was to prevent displacement of the tube. This plan consisted in having a tube with a neck and nail-head, having a small apperture at the upper extremity of the tube, through which the tears passed, and was to rest through a permanent fistula in the side of the sac. The tube was held in position by its head, which was always visible near the inner angle of eye.

(Fig. 2.)*

These plans, although extensively practiced, a few years since, have given place to more improved methods. Scarpa, an Italian, and Beer, a German surgeon, employed catgut for dilating strictures of the lachrymal duct; it was introduced through a fistulous opening in the side of the sac, and the end allowed to rest on the floor of the nostril, while the other end was fastened to the face by adhesive plaster. The portion in the duct, by absorbing the fluids of the sac, which would cause it to expand; each day the portion in the duct would be drawn through and cut off, a new piece taking the place of the old one. This method, like the preceeding one, is now obsolete.

In the early part of the last century, Anel, a surgeon of Turin, practiced dilatation of the puncta and canaliculi with a small probe, not larger than a bristle. He also devised a syringe, with a fine-pointed nozzle, to inject fluids through the puncta and canaliculi, into the lachrymal sac, and, by the force thus employed, to remove any obstruction of the nasal duct, or treat any disease of the lachrymal sac. This method of treatment, although practiced for a long time, was not satisfactory. There were many cases of stricture of the nasal duct, which the force from the syringe was insufficient to remove, and the fluid injected through the lower punctum would escape through the upper one. The objection to the probe, devised by Anel, was that it was too small and delicate to be of much service, and is,

* Dupeytren's and Walthen's tubes.

by some, regarded as dangerous to use. In certain cases, the syringe may be conveniently employed to inject the sac.

About the year 1820, Mr. Travers, a London surgeon, advocated the practice of dilating the natural passage by probes of silver-wire. This was first suggested to Mr. Travers by the strong analogy existing between the nasal duct and the urethra, and seeing the principle of dilatation practiced so successfully in strictures of the urethra. He says: "This applies to treatment of the urethra, as well as to the ductus nasalis, and it is only in cases of abscess, in which the distended and inflamed integuments threaten to give way by ulceration, that in either case it becomes necessary to deviate from it." The probes used by Mr. Travers were about five inches long, varying in size, flattened at one end, and slightly bulbous at the point. "The probe is introduced with perfect facility (by one who has an accurate knowledge of the parts) from either the upper or lower puncta lachrymalia, into the corresponding nostril, when no obstruction is offered to its passage; when the puncta is constricted, it is readily entered and dilated by a common pin, and, upon withdrawing it, by one of the smaller probes." He continues: "I have seldom met with a stricture so firm as not to yield to the full-sized probe. I am fully aware of the objection that immediately presents itself, *viz.:* that a passage so obtained is not permanent; but, by several repetitions of the operation, it is often rendered so." If, after a few days' trial, the treatment was not effectual in removing the obstruction, he would then introduce a nail-headed style, shaped to the course of the canal. This may remain in the duct for a period of twenty-four hours, after which the sac is syringed with tepid water by means of Anel's syringe. The part is then permitted to rest for twenty-four hours, after which this operation is repeated, and this plan is persisted in until the obstruction is completely overcome.

The plan devised by Mr. Travers has been practiced, with slight modifications, by an eminent surgeon of Philadelphia, Dr. Isaac Hayes, who gives the following strong testimony of its merits: "We have now employed this mode of treating obstructions of the lachrymal passages for more than twenty years, in a very large number of cases, with the most gratifying success. We are persuaded that it is the most rational and least painful mode of treatment, effecting a permanent cure, and leaving no unsightly scar, as in the case where the sac is opened, and a style or tube inserted."

21 20 19 18 17

(Fig. 3.)*

The probes used by Dr. Hayes differ slightly from those devised by Mr. Travers. They were not bulbous at the extremity, but small and rounded, and in size from No. 17 down to No. 21, wire guage.

The probe is always introduced through the lower punctum. When the punctum and duct are occluded, Dr. Hayes commences treatment by using Anel's probe, which is longer than his own, and is more conveniently manipulated. After this he commences with a No. 21, or the smallest size of his own probes, and gradually increases up to the largest, No. 17. The manner of inserting this probe is described by Dr. Hayes as follows: "The lower lid is to be drawn tense, with the left thumb applied at the outer angle of the eye, and the patient directed to look upwards. The lower punctum is thus exposed, and placed in the best position for the introduction of the probe. This instrument is then to be introduced perpendicularly to the edge of the lower lid, into the punctum, and, by a little pressure, pushed as far as the commencement of the lachrymal canal. The direction is then to be changed to a nearly horizontal position, so as to correspond to the direction of the lachrymal canal. The point being a little upwards and backwards, by gentle pressure in this direction, the probe may be pushed in, until its point passes into the sac and presses against the bone. The direction of the probe is then once more to be changed nearly to a perpendicular, and the probe to be gently pushed

* Dr. Hayes' probes for dilating the lachrymal canal, *via* the lower puncta.

down, until the stricture is passed, and the probe rests upon the floor of the nostril. No violence should be used, if it is, the membrane will be torn and injury inflicted." But if it cannot be introduced with very moderate pressure, he advises to abandon the attempt for a few days, until the irritation subsides, and then attempt it again. When you have succeeded in once introducing the probe, it is allowed to remain for "one, two, three, or even twelve hours," if it is found not to produce much irritation. He says it is rarely necessary or advisable to let it remain for a longer period than this. When the probe is withdrawn, the canal is washed out with cold water, by means of Anel's syringe. "After an interval of four, five, or eight days, to allow the tenderness to disappear, the same probe, or, if practicable, one a size larger, may be introduced. This process is to be repeated at intervals, the size of the probe being increased whenever it is practicable, until the passage has been dilated to its full extent. When this has been accomplished, it may be well to introduce the large probe a few times, at distant intervals, and inject cold water through the punctum, by means of Anel's syringe."

In the year 1851, Mr. Bowman, an English surgeon, published an account of a new operation for Epiphora, when there was eversion of the punctum. The operation consisted in slitting up the punctum and canaliculus as far as the caruncle. This operation proved so successful that it soon came into general favor. And in 1855, the same operation of slitting up the punctum and canaliculus, was practiced by Mr. Bowman, as a means of assisting in exploring the lachrymal canal, and removing obstructions from any portion of the passages. This method is now in more general use than any other, for the treatment of lachrymal obstructions. It consists in slitting up one canaliculus, generally the lower one, as far as the caruncle, and repeatedly probing the nasal duct with variously sized silver probes, of regularly increasing diameters. The probes consist of six sizes, from the smallest to the 1-20th of an inch. These probes are made of silver, or of hard rubber, which has the advantage of being more flexible than the silver, and is preferred by some on this account. They are arranged so that two sizes may be blended in one probe, one end of one size, and the opposite extremity of the next size. The punctum and canaliculus are slit open by means of fine scissors, or a small grooved director and sharp knife. There is in use, also, a small probe-pointed knife with a curved blade, which is well adapted for the purpose. The probe point of the knife is introduced into the punctum, and pushed on into the canaliculus, and the handle of the knife raised from a horizontal to a nearly perpendicular position, and as the handle is raised, the blade slits up the punctum and as much of the canaliculus as may be required, even into the sac, if necessary, which is seldom the case.

(Fig. 4.)*

The scissors spoken of, for this purpose, are also exceedingly convenient, from the fact that they enable us to regulate and limit the length of the incision. They consist of a pair of fine straight scissors, with one blade projected about one-twelfth of an inch, into a fine probe point, and the other blade not quite so long, and terminating obtusely. These scissors were devised by Dr. John Green, a distinguished ophthalmologist of St. Louis, Missouri.

After the slitting of the caruncle has been accomplished, which is neither difficult nor painful, a probe should be introduced into the canal, and gently pushed forwards, and should it come in contact with a stricture, or an obstruction of some kind, by proper care in manipulating, with one of the small sized probes, the obstruction may be passed, after which a size larger probe may be used, and so continuing to increase the size of the probe, until the canal will admit the largest sized probe throughout its entire length.

According to Mr. Bowman, in about one out of every four

* Bowman's probes for dilating, with division of lower punctum.

cases, a stricture or obstruction of some kind exists in the canaliculi, its most usual location being near the sac, but occasionally near the middle of the canaliculus. Should it exist in either of these localities, one of the smallest sized probes should be introduced, and, by care and skill, the obstruction may be passed; if it should prove to be obstinate, then slight force may be used; if, after thorough trial, it should turn out to be impervious, then it may be perforated by Mr. Bowman's guarded knife, or his canula lancet, instruments devised by Mr. Bowman expressly for this purpose. When the probe passes with perfect freedom through the canaliculus, then the larger sized probes may be passed down, and the nasal duct explored; and, if a stricture exists in the course of the sac, or nasal duct, it may be treated by the same method of gradually increasing the sizes of the probes. The canaliculus being a much smaller canal than the nasal duct, it is believed by some to be impossible to dilate a stricture of the nasal duct, through the canaliculi; so they originated the plan of slitting the canaliculus up throughout its entire extent to where it opens into the sac, thus converting it into a groove. By this means the surgeon is enabled to pass the largest sized probes or styles without spending much time, or exercising a great deal of skill. This operation frequently involves the total obliteration of the canal, and hence, as Dr. Green has said, it constitutes a mutilation, and should not be resorted to except for good and sufficient reasons. When this is done, the upper canaliculus is the one usually chosen for the operation; and it is fortunate that such is the case, as the upper one can more easily be sacrificed than the lower one.

(Fig. 5.)*

Dr. E. Williams, of Cincinnati, treats obstructions of the nasal duct by means of styles, of large graduated sizes, introduced through the upper canaliculus, by means of a free incision. The largest of these styles are one-eighth of an inch in diameter, and are worn for a period of from six weeks to three

* Dr. Williams' styles for dilating *via* the upper canaliculus, with free incision of punctum and canaliculus.

months. They are taken out every day, and the sac injected with a solution of sulphate of copper or zinc.

My friend, Dr. John Green, of St. Louis, Missouri, to whom I am in debt for much that is in this paper, has proposed a modification of Dr. Williams' treatment, by substituting the softest, most pliable lead wire, for the rigid silver styles of Dr. Williams. Dr. Green claims that the lead, being soft and pliable, adapts itself to the anatomical peculiarities of the parts: the nasal duct not being perfectly straight, but irregularly curved, or at least variable and uncertain in direction, and, hence, it is often difficult to pass a large and stiff probe; or, if passed entirely through the canal to the floor of the nostril, the pain it causes from unequal pressure being too rigid to adapt itself to all the irregularities, soon causes its removal. But the leaden style fulfills all the indications not accomplished by the silver ones:

First, it is easily introduced, and readily adapts itself to all the irregularities with comparatively little pain, and hence can be worn for a much longer period without removal. The smaller size can be more readily exchanged for the larger—than is the case with the silver ones, thus shortening the period of treatment. Experience has also shown that less dilatation is required by this method than by that in which the silver styles are used, and the injection of medicated fluids into the sac may also be dispensed with. In the use of the silver styles, the lachrymal passage is changed to conform to the style, while in the use of the leaden ones, the style adapts itself to the shape of the canal; and, frequently, when they are withdrawn, they are curved, showing the shape and course of the duct, varying in different cases, but retaining a uniformity in the various stages of treatment of the same case. At first, Dr. Green experienced difficulty in introducing the smallest sizes of these leaden styles, on account of their extreme flexibility. This led him to devise the plan of making them tubular, and having a a steel or silver stilet to fit into them. They are then rendered rigid, which facilitates their introduction—which, when accomplished, the stilet is withdrawn; and the style, remaining in place, soon adapts itself to the shape of the canal. Dr. Green uses seven sizes of these styles, numbering from No. 14 to No. 20, of the wire guage. He claims that in certain obstinate cases, where Mr. Bowman's method is not exactly suitable, because no permanent result follows the successive introduction of the probe, the stricture returns as soon as the probe is withdrawn; in cases of this class, a large sized leaden style, worn for a few days, will generally give a free passage, or, in other words, the effect on the stricture is more permanent than by the silver probes. It also accomplishes the end in view without interfering with the normal condition of the parts, much more perfectly, and in quicker time, than by the silver styles of Dr. Williams.

We have now alluded to all the principal methods of treating lachrymal obstructions. The old method of laying open the sac, is now almost obsolete, as is also the method of puncturing the integuments, and introducing a silver tube, or nail-headed style, practiced by Dupeytren, Lubbock, and Mr. Ware. But the method of treatment by dilatation, is the one which holds ascendency over all others. Each of its modifications is possessed by its own peculiar merits, and each one has advantages over the others, in certain classes of cases; and no one of them should be practiced to the exclusion of the others. But the principal of continued dilatation through the natural passage, as first practiced by Dr. Travers, and Dr. Hayes, and subsequently modified by Dr. Bowman, and others, is the best plan with which we are acquainted, for treating lachrymal obstruction, and disease of the lachrymal sac. We should be prepared to use any one of its modifications, whenever a case requiring either of these modifications presents itself. The plan which will apply to the largest number of cases, and leave them in the condition nearest resembling nature when the treatment is terminated, should be the one adopted, all things being equal. The plan of Mr. Bowman is probably more in general use than all the others put together, because it fulfills the indications in the greatest number of cases, and, all things considered, leaves the parts in a more normal condition than any other. But there are exceptional cases, in which simply slitting up the punctum, and probing according to his plan, will not suffice. The stricture returns almost as soon as the probe is withdrawn. In such cases a style, worn for several days, will be found beneficial; and we are inclined strongly to the opinion that the flexible, leaden style, as used by Dr. Green, is the one best calculated to do good in these cases. We are now using the leaden styles, as recommended by him, and in the few cases in which we have thus far made use of them, our success has been most gratifying. We have never yet used the styles recommended by Dr. Williams, for the reason that we have always practiced Bowman's method of slitting up the lower punctum, and dilating the passages with the series of probes recommended by him. Then, too, we were taught in our pupilage to believe that slitting up the canaliculi into the sac, was taking too free liberties with the parts, and was dangerous to the functions of the canaliculi, and should be practiced only in exceptional cases, and also that the use of very large styles, similar to the largest of his, was dangerous to the membranous lining of the nasal duct, from the long continued pressure of the mucous membrane against the firm, bony walls of the duct. For the same reason, we have never made use of the probes made from *laminaria digitata*, used for any obstinate strictures, the principle of which is similar to that of a sponge tent in the cervix uteri: it absorbs the fluids and swells up, causing rapid dilitation; and more, when this is removed from the passage, there is danger of its tearing, or, at least, loosening the mucous membrane from the bone which forms the wall of the nasal duct.

As regards probing the superior or inferior canaliculi, it matters but little which one is selected. A very good plan is to practice on both, as was the custom of Mr. Travers; first one, and then the other.

We have been accustomed to using the lower one, but can see no reason why it should be selected in preference to the upper, except as a matter of convenience, or where the inferior one is impassable from adhesions. It is a little more difficult to get at the superior one, especially where the eye is not very prominent; neither is it easy to stretch the canaliculus in the proper direction, in order to slit it, as with the lower one.

In conclusion, we would simply remark that, all things considered, the system of Mr. Bowman presents advantages over all others. The interference of the natural conditions of the parts is so slight, and the results on the whole so perfect, and the pain and inconvenience of the patient so trivial, and the time occupied in the treatment probably as short as in any other, that by far the large majority of cases may be easily and permanently cured.

## Figures and Tables

**Fig. 1. f1:**
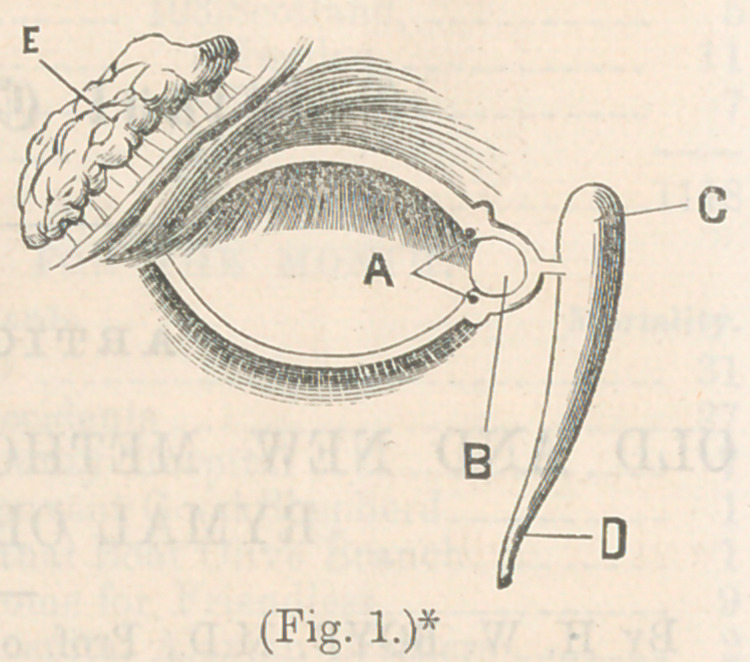


**Fig. 2. f2:**
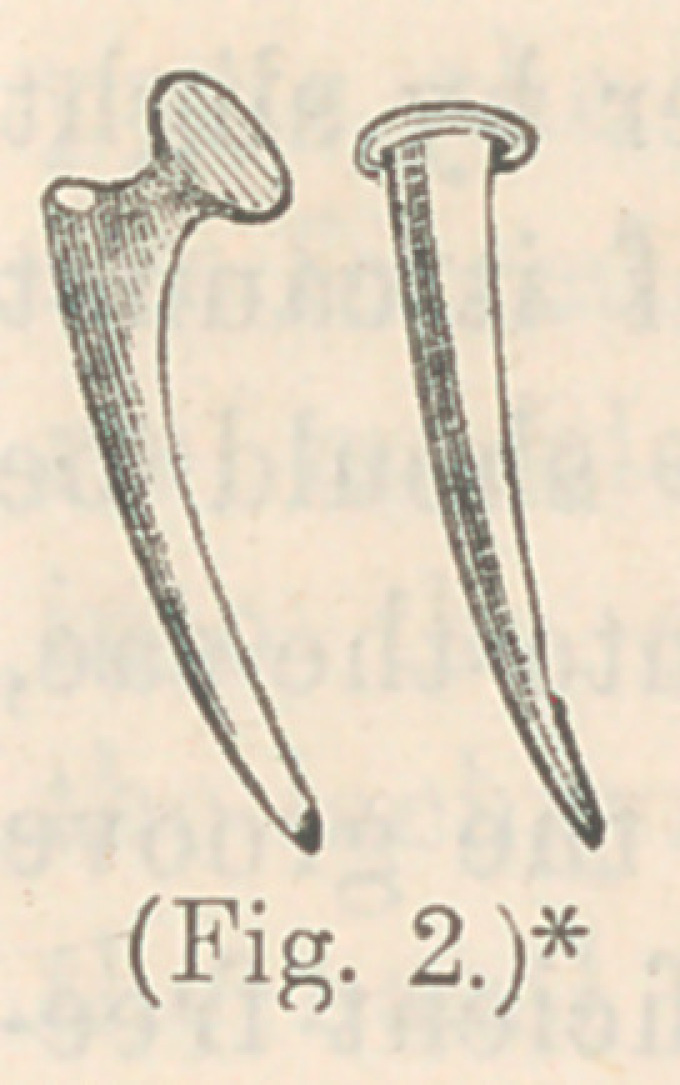


**Fig. 3. f3:**
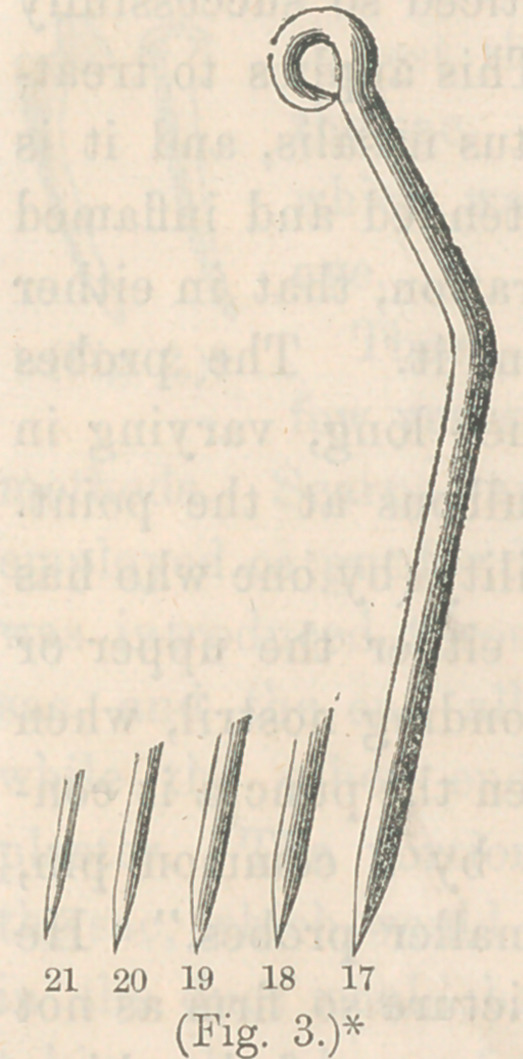


**Fig. 4. f4:**
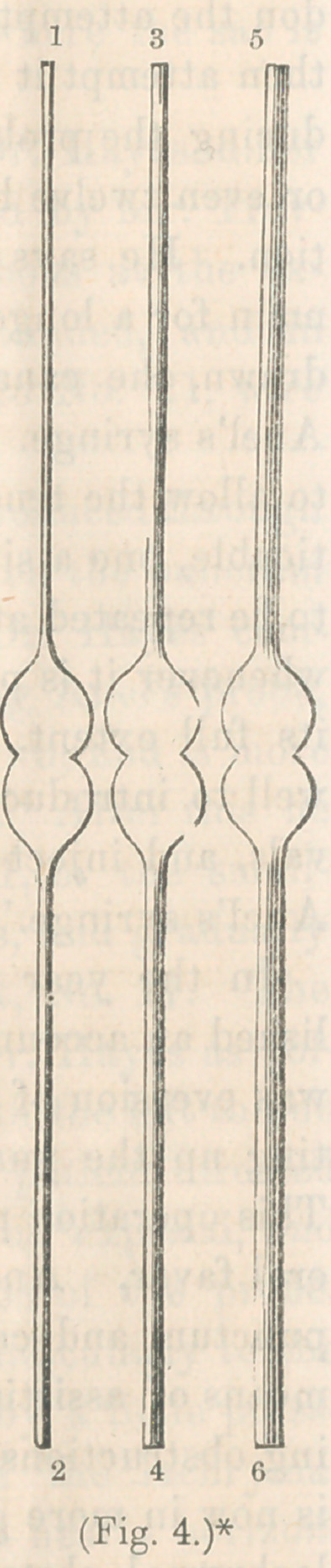


**Fig. 5. f5:**